# Accelerated Particle Separation in a DLD Device at Re > 1 Investigated by Means of µPIV

**DOI:** 10.3390/mi10110768

**Published:** 2019-11-11

**Authors:** Jonathan Kottmeier, Maike Wullenweber, Sebastian Blahout, Jeanette Hussong, Ingo Kampen, Arno Kwade, Andreas Dietzel

**Affiliations:** 1 Institute for Microtechology, TU Braunschweig, 38124 Braunschweig, Germany; a.dietzel@tu-braunschweig.de; 2 Center of Pharmaceutical Engineering (PVZ), TU Braunschweig, 38106 Braunschweig, Germany; m.wullenweber@tu-braunschweig.de (M.W.); i.kampen@tu-braunschweig.de (I.K.); a.kwade@tu-braunschweig.de (A.K.); 3 Institute for Particle Technology, TU Braunschweig, 38104 Braunschweig, Germany; 4 Institute for Fluid Mechanics and Aerodynamics, TU Darmstadt, 64287 Darmstadt, Germany; sebastian.blahout@ruhr-uni-bochum.de (S.B.); hussong@sla.tu-darmstadt.de (J.H.)

**Keywords:** microfluidics, deterministic lateral displacement, Reynolds number, particle image velocimetry, size-dependent fractionation

## Abstract

A pressure resistant and optically accessible deterministic lateral displacement (DLD) device was designed and microfabricated from silicon and glass for high-throughput fractionation of particles between 3.0 and 7.0 µm comprising array segments of varying tilt angles with a post size of 5 µm. The design was supported by computational fluid dynamic (CFD) simulations using OpenFOAM software. Simulations indicated a change in the critical particle diameter for fractionation at higher Reynolds numbers. This was experimentally confirmed by microparticle image velocimetry (µPIV) in the DLD device with tracer particles of 0.86 µm. At Reynolds numbers above 8 an asymmetric flow field pattern between posts could be observed. Furthermore, the new DLD device allowed successful fractionation of 2 µm and 5 µm fluorescent polystyrene particles at *Re* = 0.5–25.

## 1. Introduction

**Figure 1:**
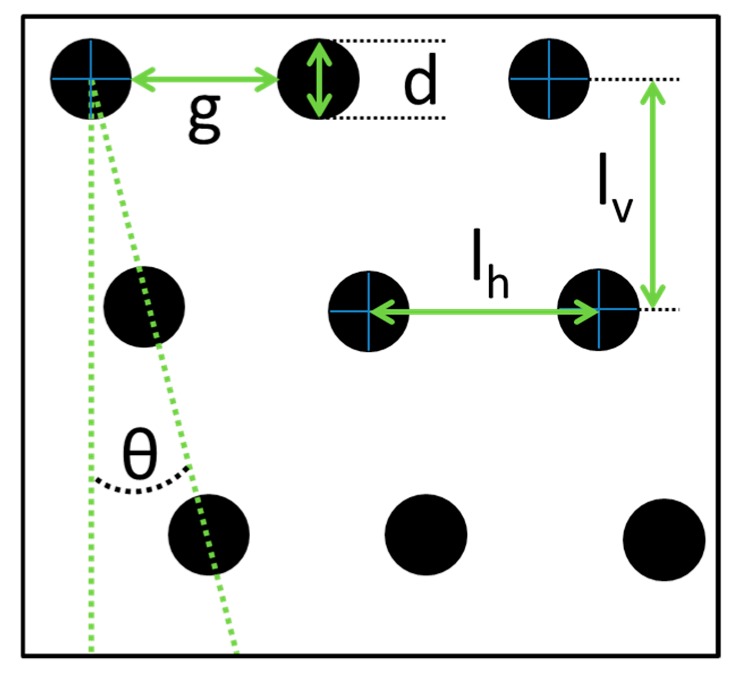
Illustration of the design parameters for a deterministic lateral displacement (DLD) array, with gap between posts (*g*), diameter of posts (*d*), row shift angle (*θ*) defining the period and the row shift fraction, as well as the horizontal and vertical center-to-center distance (*I_h_* and *I_v_*).

Pharmaceutical drugs with poor water solubility exhibit a size dependent resorption. Therefore, only a small range of particle sizes is suitable to ensure maximal efficacy of a specific particulate pharmaceutical drug. During the process of formulation, a uniform size within a narrow size distribution is sought [1]. Another application of high-throughput microfluidic systems is the size dependent up-concentration of biological materials that behave as particles [2]. To address these tasks, different methods have been implemented in microfluidics, such as fractionation in a spiral microchannel, multi-orifice flow fractionation (MOFF) and deterministic lateral displacement (DLD) [3,4]. In contrast to spiral channels and MOFF devices, DLD arrays allow separating particles of small size differences down to 0.3 µm, in specially designed arrays even down to 10 nm, within a dynamic range of particle sizes around five inside a single array [5,6]. A DLD fractionation device array consists of an array of tilted microposts with a gap between posts (*g*), post diameter (*d*) and period (*N*) as illustrated in Figure 1. The period (N), tilt angle (*θ)* and row shift fraction (*ε*) are related as described in Equation (1) [2]. The flow between two posts is divided into a number of streamlines given by the array period each carrying the same volumetric flow rate. Due to the no slip condition at the walls and posts, the streamlines next to the posts are wider compared to the streamlines in the middle between two posts [5]. As long as the particles are smaller than the first streamline (in other words: Are below the critical diameter), they will follow this streamline through the array and will therefore not be displaced. Particles with a diameter higher than this streamline will bump against the posts, thereby the hydrodynamic center of the particles will change the streamline and the particle will move in the so-called displacement mode through the array. The critical diameter (*D_c_*) can be calculated for lower *Re* using Equation (2) as an empirically founded relation [2]. In standard lower throughput operation (*Re* ≤ 1), using spherical particles and cylindrical posts, *D_c_* is only based on the array geometry such as tilt angle and gap between posts [2,5]. To increase the range of particle sizes fractionated inside one array, it can be segmented changing either the gap between posts (*g*) or the tilt angle (*θ*) [2,5]. At high throughput (*Re* > 9) stationary vortices form behind the posts resulting from inertial forces, which change the fractionation properties [5,7,8,9].

*ε* = 1/*N* = tan (*θ*) (1)

*D_c_* = 1.4·*g*·*ε*^0.48^ (2)

In addition to using geometry or flow control, the critical size for fractionation can also be adjusted by electrokinetic effects such as electrophoresis, electro-osmosis or electrostatic effects based on the ion concentration of the buffer solution [10,11]. In addition, a stretchable array was fabricated to alter *D_c_* by mechanical stress applied to the array [12]. 

High-throughput applications of DLD arrays include the up-concentration of cycling tumor cells from blood samples whilst removing the blood plasma. For this purpose, stacked arrays of triangular posts were operated at a flow rate up to 10 ml/min (*Re* > 40) [13]. In this case, a stable separation was observed up to higher Reynolds numbers (*Re* = 40) [13]. Furthermore, the fractionation characteristics and visualization of flows at higher Reynolds numbers was investigated using 70× up-scaled arrays with circular posts with a diameter of 0.675 mm [7,8,14]. The flow around the posts was visualized using 100 µm particles in a range of 2 ≤ *Re* ≤ 30. With high-speed particle trajectory recording, stationary vortices behind posts have been observed for *Re* > 16 [8]. More recently, a study about miniaturized arrays with a gap between posts (*g*) of 50 µm and 30 µm, operated up to *Re* = 50 (*g* = 50 µm) and *Re* = 35 (*g* = 30 µm) has been reported [9]. By means of high-speed trajectory recording in the smaller array, a shift of the particle positions at the outlets was observed for particles at a diameter around half of the gap (*g* = 30 µm, *d* = 15 µm) explained by emerging vortices behind the microposts which could not be directly visualized [9]. To minimize the vortices behind posts, also airfoil shaped posts were investigated [15]. The experimental results using airfoil shaped posts and circular posts at higher Re were compared to COMSOL multiphysics simulations [9,15]. Up to now, all published experimental works in the field of high-throughput DLDs rely on high-speed particle trajectory recording at the end of the array and observation of the fractionation at the outlets. The fractionation results are typically compared with simulations using computational fluid dynamic (CFD) simulations with ANSYS FLUID and STAR-CCM+, based on the Lattice Boltzmann method; either as a standalone to solve the velocity field or coupled with FEM using the immersed boundary method to account for fluid–particle interactions [5,14,16,17,18,19]. However, up to now, there is no direct experimental evidence of the asymmetry of the flow velocity distribution induced by vortices behind the microposts, which would require microparticle image velocimetry (µPIV) inside a DLD array at *Re* > 1. µPIV is based on picture evaluation using two frames at a time difference adjusted to the particle velocity [20]. The flow field results from cross-correlation of consecutive interrogation windows at identical positions. The amount of details that can be resolved depends on the particle seeding density [20]. To enable such experiments, we fabricated a pressure stable Si/glass DLD system, with a transparent lid allowing µPIV and other optical measurements. The array was segmented to allow fractionation with multiple sizes by changing the tilt angle. Tracer particles must provide sufficient fluorescent signal for µPIV but must be small enough to follow the flow in vortices forming behind the posts. For the first time, µPIV measurements in DLD devices at elevated Re with seed particles smaller than 1 µm could be directly compared with CFD simulations.

## 2. Materials and Methods

### 2.1. Segmented DLD Design

**Figure 2:**
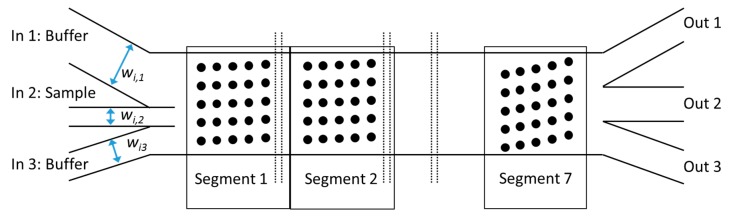
Illustration of the segmented array. The sketch is not to scale and in the real device, there is no gap between segments. The expected particle sizes in each outlet are as follows. Out 1: 0 µm (non-displaced) to 3 µm, Out 2: 3.8–5.3 µm, Out 3: > 6.0 µm.

To allow a wider range of particle sizes to be fractionated, a segmented array was designed. Either the gap between posts or the tilt angle can be altered between segments to stepwise increase *Dc*. In this work, we focus on the latter, because changing the gap between posts will lead to a non-uniform fluidic resistance and the segments with a smaller gap could easily clog. We implemented seven segments each with posts of a constant size *d* = 5 µm (defined in the lithographic mask as 10 µm) positioned with a pitch of *l_H_* = *l_V_* = 20 µm as illustrated in Figure 2. The tilt angle was changed between the segments according to Table 1 from 1.0° up to 6.7° to increase *Dc* in steps of 0.8 µm from 3.0 to 7.5 µm. Each segment is designed to displace the particles by 80 µm. The two buffer inlets in Figure 2 are included to provide a focusing of the inlet sample to a defined start position. The widths of the inlet channels (*w_i_*) are different and the individual flow rates of the syringe pumps have to be adjusted to achieve uniform lateral flow. Starting from the desired velocity *v* inside the array in relation to the simulations, the sum of the flow rates for all three inlet channels *Q_tot_* was calculated according to Equation (3). For the calculation, the height (*h*) and width of the complete array, which is not covered by posts (*w_arr_*), is taken into account. Therefore, the ratio of the horizontal center-to-center distance (*l_h_*) and the gap between posts (*g*) was used to define the free width of the complete array (*w* = 730 µm). The individual volume flow rate for the individual inlet channel *Q_i_* results from the sum of all inlet channel widths and the individual width *w_i_* according to Equation (4) for uniform lateral flow without secondary flows perpendicular to the main flow.

(3) $$Q_{tot}=v\cdot A=v\cdot w_{arr}\cdot h=v\cdot 730\;µm\cdot \frac{g}{l_{h}}\cdot h$$

(4) $$Q_{i}=\;\frac{Q_{tot}\cdot w_{i}}{\sum w_{i,j}}$$

### 2.2. Fabrication of the DLD Devices

**Figure 3:**
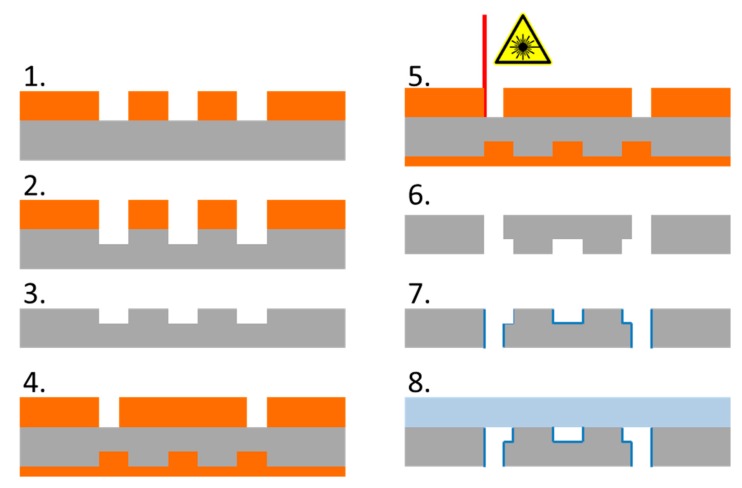
Schematic illustration of the microfabrication steps for a Si/glass DLD array. The steps are: 1. Photolithographic structuring of the resist for micropost arrays and connection channels; 2. Dry etching of silicon (Bosch Process); 3. Stripping of the resist; 4. Filling of the arrays with resist, structuring the resist for fluidic vias; 5. Fs-laser cutting of the vias; 6. Stripping the resist; 7. Deposition of 200 nm oxide layer; 8. Anodic bonding of glass and silicon.

**Figure 4:**
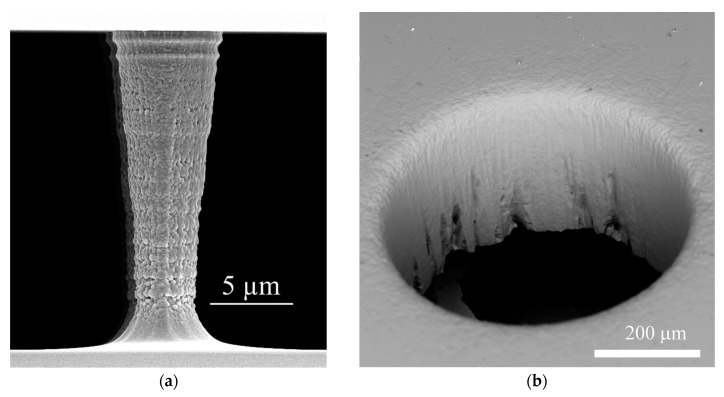
Scanning electron micrographs showing the post arrays and fluidic connections. (**a**) Detailed view of the micropost array showing the tapered profile. (**b**) Via for fluidic connections created by fs-laser induced plasma cutting.

In order to allow for high throughput, the arrays were made of silicon and glass, sealed by anodic bonding as described earlier [21] to resist high pressures. The microfabrication steps for high pressure DLD devices are illustrated in Figure 3. The microstructures for the post arrays and fluidic channels are transferred into photoresist by photolithography before dry etching the silicon in a Bosch process (using STS Multiplex ICP, Surface Technology Systems, Newport, UK) to a depth of 20 µm. Figure 4a shows the scanning electron micrograph of a single post. The fluidic vias in silicon for fluidic connections were fabricated using a fs-laser induced plasma cutting process that can produce almost perpendicular via sidewalls [22]. The wafer was placed inside a holder and immersed in a constant water flow to generate a moving thin water film on top of the surface to facilitate plasma agitation and heat transport. Before cutting, the arrays were covered with a photoresist to prevent destruction of the microposts and uptake of particles generated by laser cutting. A scanning electron micrograph (Figure 4b) shows a fluidic via with a sidewall angle of 88°. To lower agglomeration around posts and improve the filling of the device, a 200 nm PECVD (plasma enhanced chemical vapor deposition using STS 310 PC from STS instruments) oxide layer was applied which lowers the surface tension. The arrays were finally sealed using anodic bonding at increased voltage (700 V instead of the typically used 400 V) to allow a strong bond even in the presence of the insulating oxide layer. 

To further analyze the dimensions of an array used in µPIV measurements in detail, we broke one of the used arrays with a diamond pen to define the breaking point. A SEM micrograph of two posts is depicted in Figure 4a and shows the tapered shape of a single post (*d_min_* = 3.59 ± 0.33 µm; *d_max_* = 6.56 ± 0.43 µm; *d_avg_* = 5.08 ± 1.54 µm and *h* = 19 µm). In the further considerations, we therefore assumed an averaged post diameter of *d* = 5 µm, which leads to a gap of *g* = 15 µm and a height of *h* = 19 µm. Figure 4b shows defects at the bottom of the vias after laser induced plasma cutting. These are a result of the non-uniform resist coating caused by the hydrophobic nature of the photoresist. 

### 2.3. Flow Control Setup

As described in Section 2.1, the array is fed using three inlet channels, which were connected to three NEMESYS syringe pumps. Inlet 1 was connected to a mid-pressure pump equipped with a 10 mL stainless steel syringe. The two low-pressure modules for inlets 2 and 3 feeding the sample and smaller buffer channels were each equipped with a 2.5 mL glass syringe. The DLD chip was placed between an aluminum plate (for fixation) and a PMMA holder (with fluidic inlets) to connect the array using standard high-pressure liquid chromatography (HPLC) connectors. 

Table 2 shows the flow rates in the experiments (*Q_1_*, …, *Q*_3_) and the resulting average velocities in the inlets (*v_in_*) which are equal for all inlets thus avoiding secondary flows perpendicular to main flow direction. Furthermore, in Table 2 the average velocity inside the array (*v_arr_*) as well as the estimated Reynolds number (*Re*) is shown. The inlet velocity inside the inlet channels was calculated according to Equation (5) using *Q_tot_*, the height of the array (*h*) and the sum of the widths of the inlet channels (*w*_*i*,1_ + *w*_*i*,2_ + *w*_*i*,3_) as described in Section 2.1. *v_arr_* was calculated according to Equation (6). The effective width of the array not covered by posts is given as *w_arr_*. Therefore, the designed width of the complete array (*w* = 730 µm) was multiplied with the ratio of the horizontal center-to-center distance of the posts (*I_h_*) and the gap between the posts (*g*). The Reynolds number was calculated, using *v_arr_* and standard values for water at 25 °C for the density (*ρ* = 1000 kg/m^3^) and viscosity (*η* = 10^−3^ Pa∙s) according to Equation (7). The hydrodynamic length was taken as the gap between the posts.

For particle fractionation experiments at different Reynolds numbers, 2 µm and 4 µm fluorescent green labeled polystyrene particles (exc. 470 nm, emm. 505 nm, FluoroGreen Thermo Scientific, bought via Distrilab Particle Technology, Leusden, The Netherlands) were suspended in a 1% (*w*/*v*) Pluronic F-127 solution and injected through the sample inlet.

(5) $$v_{in}=\frac{Q_{tot}}{h\cdot w_{in}}=\frac{Q_{1}+Q_{2}+Q_{3}}{h\cdot \left(w_{i,1}+w_{i,2}+w_{i,3}\right)}$$

(6) varr=Qtoth·warr=Qtoth·730 µm·gIh

(7) $$Re=\frac{\rho \cdot v_{arr}\cdot g}{\eta }$$

### 2.4. µPIV Setup

**Figure 5:**
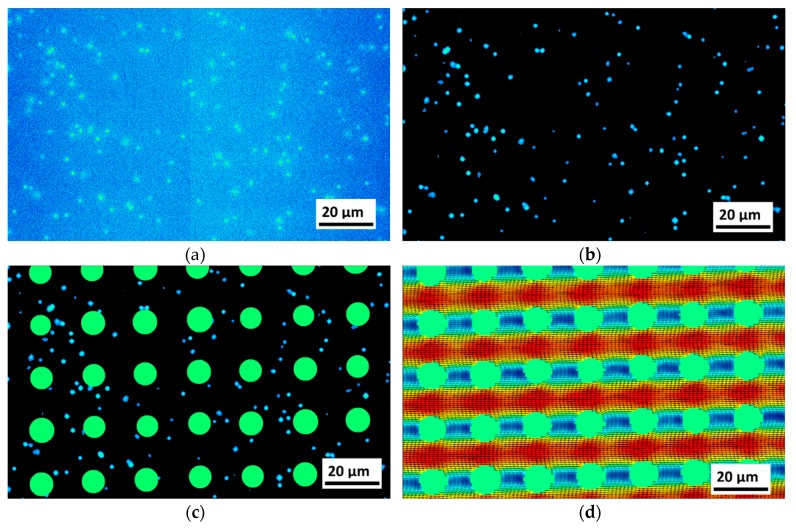
Illustration of the image processing steps (example image taken for *Re* = 7.62): (**a**) The raw camera fluorescence image. In the center of the image, the brightness level slightly but abruptly changes, which is an artifact induced by the camera technology and does not cause problems with the image correlation procedures. (**b**) Image after post processing. (**c**) Image with post areas masked. (**d**) The final µPIV result of the measurement set consisting of 1500 frames. All Images show precisely the same area.

µPIV measurements were performed utilizing an upright EPI-fluorescence microscope (Nikon Eclipse LV100) with an objective lens of *M* = 50 magnification and a numerical aperture of NA = 0.6 (50× Nikon CFI60 TU Plan Epi ELWD, Nikon, Düsseldorf, Germany) leading to a correlation depth of 7.03 µm according to the calculation described by Olsen and Adrian [23]. Since µPIV measurements utilize volume illumination, the fluorescence signal of defocused particles has a contribution to the cross-correlation result. The correlation depth indicates the distance relative to the focal plane at which this contribution occurs and is therefore a measure for the spatial averaging of the velocity field along the channel height [20,23]. Images are recorded with a double-frame CCD camera (LaVision Imager pro SX, LaVision GmbH, Göttingen, Germany). Laser and camera are triggered and monitored with the commercial software DaVis 8.4 (LaVision GmbH, Göttingen, Germany). The camera has a resolution of 2058 pixel (px) × 2456 px, which results in a field of view of 0.227 mm × 0.190 mm. The minimal size of tracer particles for PIV measurements is limited because sufficient light from the particle has to reach the camera. Fluorescent (emm. 532 nm, exc. 605 nm) polystyrene particles of 0.86 µm ± 0.04 µm in diameter (Fluoro-Max R900 from Thermo Scientific) were still providing sufficient light. Prior to injecting, particles were suspended in the carrier fluid, which is a mixture of water and 1% (w/v) Pluronic F-127. Before injecting particle suspensions, the array was flushed for 30 min with the carrier fluid. Tracer particles are excited by the beam of a double-pulsed, dual-cavity Nd:YAG laser (Litron Nano S 65-15 PIV, Litron Lasers, Rugby, UK, emm. 532 nm), which is coupled into the microscope. Images for µPIV measurements were taken using the double frame exposure mode with a short time interval (*dt*) between *dt* = 0.6 µs and 50 µs depending on the flow rate. The time interval was manually set to ensure a particle shift between the double frames of ~10 px. Double frame images ware taken at a rate of 6.5 fps. Image processing steps applied to the raw fluorescence image are depicted in Figure 5. Image processing (tools provided by DaVis 8.4) is used to isolate the particle fluorescence from background noise of the camera. Furthermore, stationary agglomerations around posts were removed before vector evaluation. Background noise was further reduced by subtracting the sliding Gaussian average of 19 frames. Second, the salt and pepper noise was reduced using a band pass filter removing everything below 5 px and above 12 px isolating the particle size signal which is in the range of 7 to 11 px. To completely isolate the particle signal, it was necessary to set all intensities below a defined value (3 counts) as provided by the software, to zero, as illustrated in Figure 5b. In a next step, the areas with posts obtained from white light images were manually masked out. 

Vector calculation is achieved using the shift of a correlation peak inside interrogation windows in two subsequent images, as described elsewhere [20]. In this work, we chose a so-called multi-pass procedure with calculation passes using each a different interrogation window size and start position. For the first pass, the interrogation window (96 px × 96 px) covered the size of a micropost to get a first fast overview. The interrogation windows of the second and third pass was chosen to be at 32 px, to resolve details around the posts. Between the second and third pass, the start position of the interrogation windows was changed. Each measurement contained 1500 images and was evaluated using the sequential sum of correlation, as provided by the software, which summarizes the average of all cross-correlation results in the correlation plane to compensate for the relatively low particle seeding density.

### 2.5. Simulation

**Figure 6:**
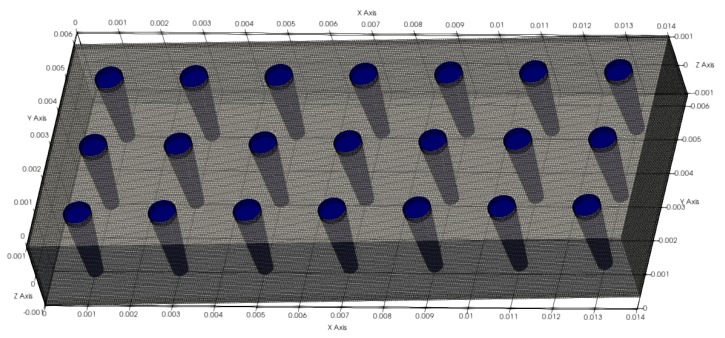
Simulation domain with tapered microposts (dimensions in 10^−2^ m).

Computational fluid dynamic (CFD) simulations were performed using the open-source software package OpenFOAM. The simulations were carried out in a three-dimensional fragment of the microsystem, consisting of seven post rows with three posts per row at a height of 21 µm (Figure 6). The microposts have a conical shape with a diameter of 6.5 µm at the top and 3.5 µm at the bottom. Density (*ρ*) and kinematic viscosity (*ν*) of the Newtonian fluid were assumed as *ρ* = 1 g/cm^3^ and *ν* = 0.01 cm^2^/s respectively. Fluid flow is achieved by defining an inlet velocity and a pressure drop in the x-axis direction. The inlet velocities v_inlet_ (free approaching flow) were calculated according to Equation (8) by multiplying the average velocity *v* between posts by the ratio of the gap *g* to the center-to-center distance *I_h_*.

(8) $$v_{inlet}=v\cdot \frac{g}{I_{h}}$$

Inlet velocities are listed with their corresponding Reynolds numbers in Table 3. In order to obtain uniform velocity profiles normal to the flow direction (y-axis), cyclic boundary conditions were assigned to the upper and lower patches. Because of the pressure drop it was not possible to define cyclic boundary conditions in x-axis direction. This problem was solved by using a function that maps the fully developed velocity profile, which is generated at the outlet, to the inlet patch. In z direction, no-slip boundary conditions were assumed. The Navier-Stokes equations are solved using the PISO algorithm which has the ability to solve transient, incompressible flows. The reason for using this transient solver is that it is also used in the CFD-DEM (computational fluid dynamics-discrete element method) coupling, in order not to get differences in the flow field due to a solver change when introducing particles into the system. The flow field was discretized using Finite Volume Discretization. A laminar flow was assumed. The time step was chosen small enough that a Courant number of 0.5 was not exceeded. The calculation period was sufficient for the flow to fully develop and not change over time. At the velocities given here, the state of the flow field remains in the stationary range.

As a post-processing step, velocity and vector fields at the middle-z-position of the channel were visualized using the software ParaView. Since the correlation depth in the µPIV measurements was 7.03 µm, a layer of the simulation domain with a thickness of 7 µm with layer center at *z* = 0 (being the middle position in the channel) was cut and averaged over the layer thickness to enable a comparison to experimental results. For *Re* = 1.52 this process was repeated for different z-positions in 2.5 µm steps in order to compare the flow profile over the height of the microsystem. For getting the maximum velocities, the global maximum of these averaged layers was determined. For the average velocities between the posts, the mean value of the velocity profile between two adjacent posts was taken.

## 3. Results and Discussion

### 3.1. Simulations

**Figure 7:**
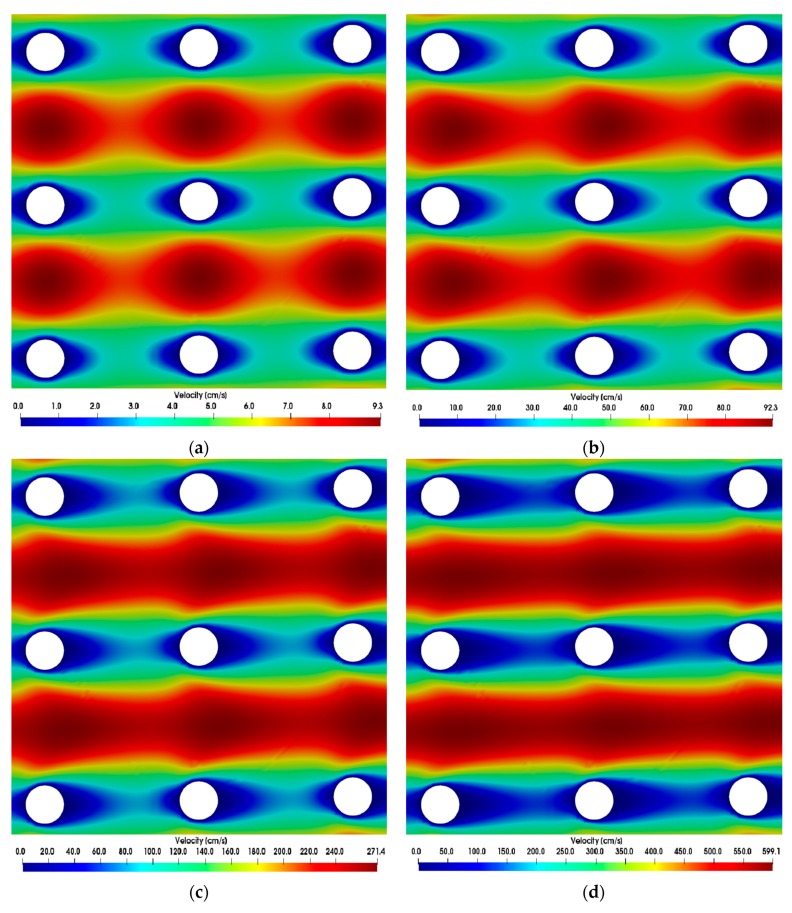
Simulations show emerging asymmetric flow between posts at different Reynolds numbers. The direction of flow is from left to right. Color scales refer to the velocity magnitude. (**a**) *Re* = 0.76, (**b**) *Re* = 7.63, (**c**) *Re* = 22.86, (**d**) *Re* = 50.

**Figure 8:**
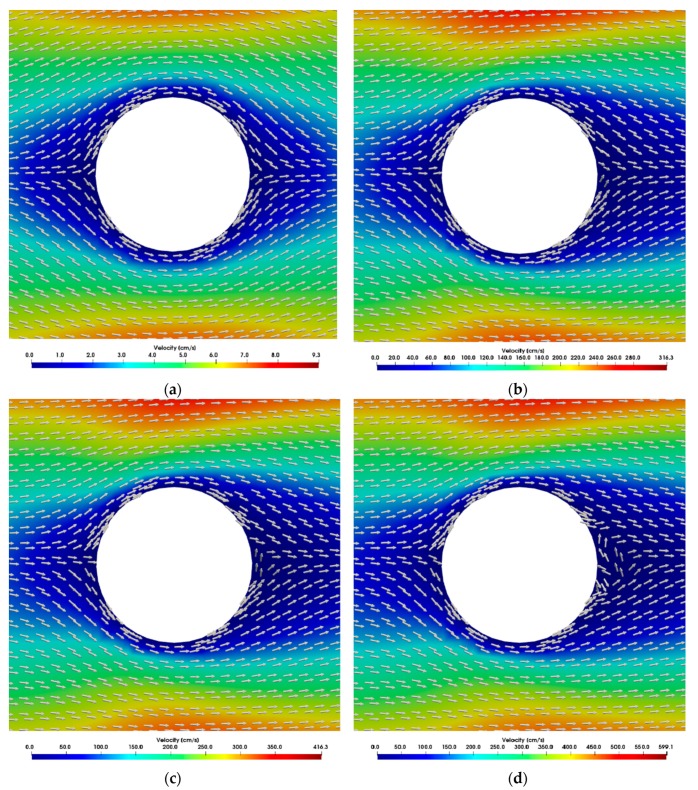
Simulated vector fields between posts at different Reynolds numbers. Static vortices begin to arise with a growing Reynolds number. Color scales refer to the velocity magnitude. The direction of flow is from left to right. (**a**) *Re* = 0.76, (**b**) *Re* = 26.67, (**c**) *Re* = 35, (**d**) *Re* = 50.

The wake behind the posts observed in the simulations was compared for different Reynolds numbers. The flow around the DLD microposts can be compared to the flow around a cylinder, which has often been investigated in the literature [24,25]. However, adjacent microposts can also influence the flow. In literature, attached flow behind a cylinder was proven for *Re* < 5, while for larger *Re* up to 40 a fixed pair of vortices was detected [24]. With further increase in Reynolds number, it was found that the vortices became unstable and began to detach alternately (Karman vortex street). The transition range to turbulence is 150 ≤ *Re* < 300 [24]. Considering the flow around the posts in the microsystem, a similar behavior can be observed. Whilst at Reynolds number up to *Re* = 1 the wake behind the posts is nearly symmetrical to the stagnation point flow in front of the posts, with growing Reynolds numbers the wake elongates which is expressed in a growing asymmetry (when mirrored with the flow in front of the posts) as shown in Figure 7. The relative velocities in different z-planes did not change during the simulation of tapered posts and are therefore not displayed. At Reynolds numbers of about 50 the wake of the trailing edge passes almost directly into the leading edge flow of the following post. This means that the microsystem is continuously traversed along the direction of flow by lines in which the fluid and thus the particle velocity is strongly decelerated. Vector fields show that at Reynolds numbers up to around 1 the fluid stream splits up in front of the posts and flows back together directly behind the posts (Figure 8a). At Reynolds numbers around 20, a static vortex begins to form behind the posts, which grows with the Reynolds number and causes a stall (Figure 8b–d). The flow remains stationary at *Re* ≤ 50 and no vortex shedding was observed.

### 3.2. Comparison between Simulations and µPIV Results

**Figure 9:**
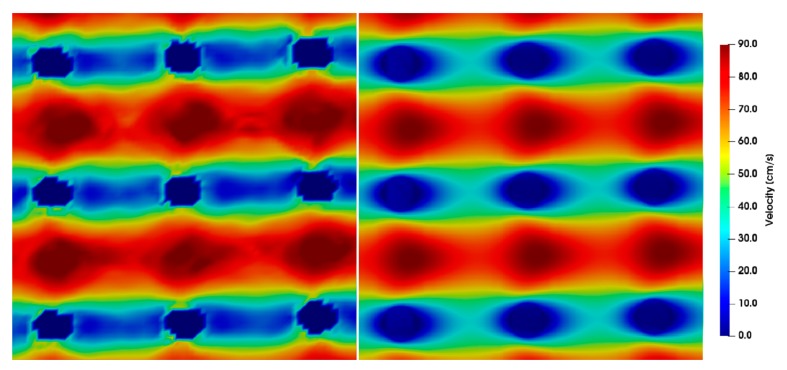
Microparticle image velocimetry (µPIV) measurements (**left**) compared to simulations (**right**) showing the velocity field around posts at *Re* = 7.62. The direction of flow is from left to right, the pitch between the posts is 20 µm at a time difference between double frames of *dt* = 3 µs.

**Figure 10:**
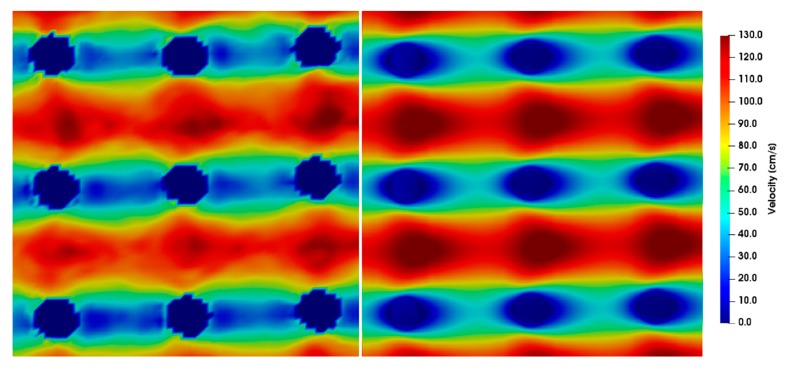
µPIV measurements (**left**) compared to simulations (**right**) showing the velocity field around posts at *Re* = 11.43. The direction of flow is from left to right, the pitch between the posts is 20 µm and the time difference between double frames is *dt* = 1 µs.

**Figure 11:**
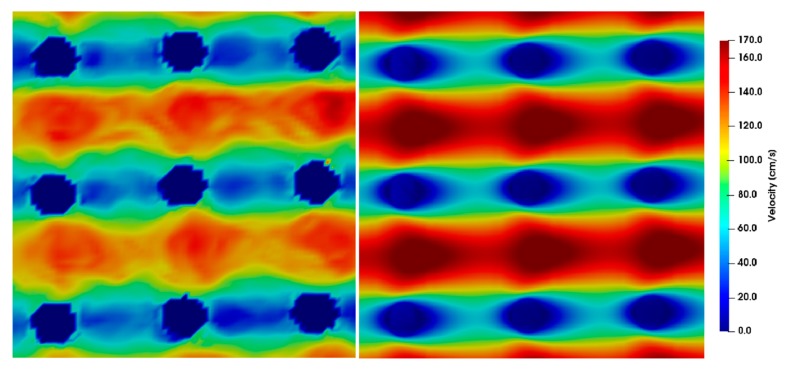
µPIV measurements (**left**) compared to simulations (**right**) showing the velocity field around posts at *Re* = 15.24. The direction of flow is from left to right, the pitch between the posts is 20 µm and the time difference between double frames is *dt* = 1 µs. The µPIV measurements do not attain the maximum velocities achieved by the simulations.

**Figure 12:**
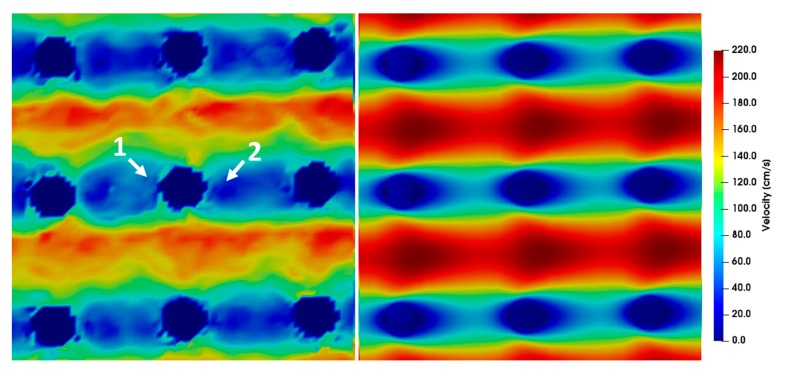
µPIV measurements (**left**) compared to simulations (**right**) showing the velocity field around posts at *Re* = 19.05. The direction of flow is from left to right and the pitch between the posts is 20 µm and the time difference between double frames is *dt* = 0.8 µs. For a better highlighting of the asymmetry, two arrows are inserted into the picture. The velocity before the post is higher (arrow 1) than the velocity after the post (arrow 2). Furthermore, the µPIV measurements do not attain the maximum velocities achieved by the simulations.

**Figure 13:**
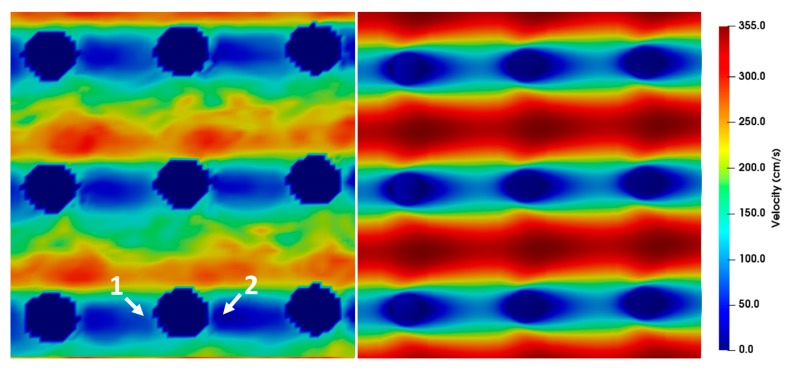
µPIV measurements (**left**) compared to simulations (**right**) showing the velocity field around posts at *Re* = 30.48. The direction of flow is from left to right and the pitch between the posts is 20 µm and the time difference between double frames is *Δt* = 0.6 µs. For a better highlighting of the asymmetry, two arrows are inserted into the picture. The velocity before the post is higher (arrow 1) than the velocity after the post (arrow 2). Furthermore, the µPIV measurements do not attain the maximum velocities achieved by the simulations.

**Figure 14:**
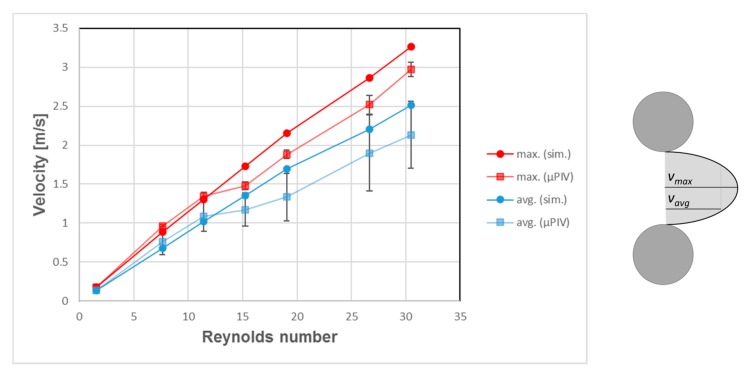
Velocities (maximum and average between posts) depending on Reynolds number at median z layer. The simulation data shows a linear correlation between Reynolds number and both maximum and average velocity. The µPIV data, however, show that velocities do not continue to grow in proportion at Reynolds numbers higher than 15.

The simulation results for different Reynolds numbers were compared to the µPIV results at position *z* = 0 (Figure 9, Figure 10, Figure 11, Figure 12 and Figure 13). For each µPIV measurement, a white light image was taken to obtain the position of the microposts. Therefore, the posts were manually marked and masked out. Asymmetries between the area in front of the post and behind the post appear in the simulations as well as in the experiments at *Re* > 1. These asymmetries grow with increasing Reynolds number, which is especially visible in the simulations. At Reynolds numbers up to around 10, the simulation velocity fields resemble the µPIV results. At higher Reynolds numbers, a difference is notable between µPIV and simulations (see the color code in Figure 11, Figure 12 and Figure 13). The µPIV velocities are slightly lower when compared to the ones obtained in the simulations. In addition, the flow asymmetries in Figure 12 and Figure 13 are difficult to detect. Therefore, we have highlighted the differences before and after the posts. It is possible that vortices form also in z-direction, but our measurement equipment is not capable to detect vorticities along the channel height. The maximum and average velocities in flow direction at the median z layer between posts that are direct neighbors in y direction (perpendicular to the flow direction) as obtained from µPIV experiments and from CFD simulations are shown in Figure 14. The velocities obtained in the simulations linearly increase with the Reynolds number but the velocities from µPIV are slightly lower at Reynolds numbers higher than 15. We assume that the major reasons for the deviations are agglomerations of particles around posts in sections of the array located outside the field of view, which could not be monitored simultaneously. Other sources of deviation could be slight variations in the masking during post-processing of µPIV measurements that affect the average velocities between posts. Furthermore, the average velocity from the simulation is obtained inside a 7.03 µm thick layer at the median z-position, in which posts are assumed with perpendicular sidewalls and gaps are therefore constant. The layer thickness was adjusted to the experimental correlation length of 7.03 µm. The real silicon microposts are tapered, as could be seen in the SEM micrographs, we expect a varying gap and flow velocities changing with depth.

**Figure 15:**
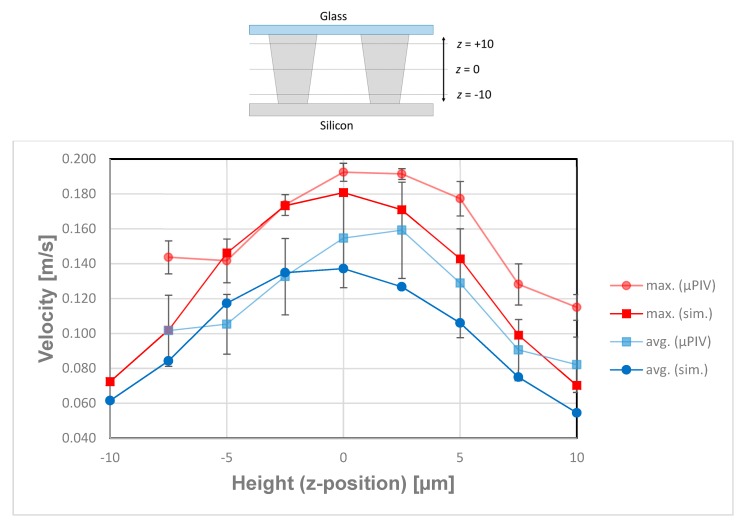
Maximum and averaged velocities at *Re* = 1.52 between neighboring posts obtained by µPIV and simulations for varied focal depth (z-axis within the array). For this evaluation, for every layer, four rectangle areas between posts were chosen out of the µPIV velocity fields and the velocity maxima and mean values of these four areas were averaged.

To gain information about the flow profile along the z-direction, we conducted µPIV measurements at different z-positions in the area between neighboring posts. Five areas between posts were manually selected in which the average and maximum velocities were calculated for different z-heights and compared to the simulation of tapered posts. The resulting parabolic flow profiles at *Re* = 1.52 are depicted in Figure 15. It is notable that the velocity maximum in the µPIV experiments is shifted 2.5–5 µm in direction of the top cover of the microsystem. Since the posts are tapered as shown in Figure 4a, deviations of the velocity maximum value and the maximum position between µPIV and simulation scan be explained by the height dependent gap between the posts. The channel bottom position (*z* = −10) was obtained by focusing particles after allowing them to settle for 30 min (density particles 1.05 g/cm^3^, density water 1 g/cm^3^). We believe that the velocity shift and higher values of maximal velocities are caused by the tapered profile of the posts, which leads to a changed partial pressure along the z-direction of the array. The flow counteracts the pressure and thereby the velocity is modified in comparison to the simulation. 

### 3.3. Fractionation Results

**Figure 16:**
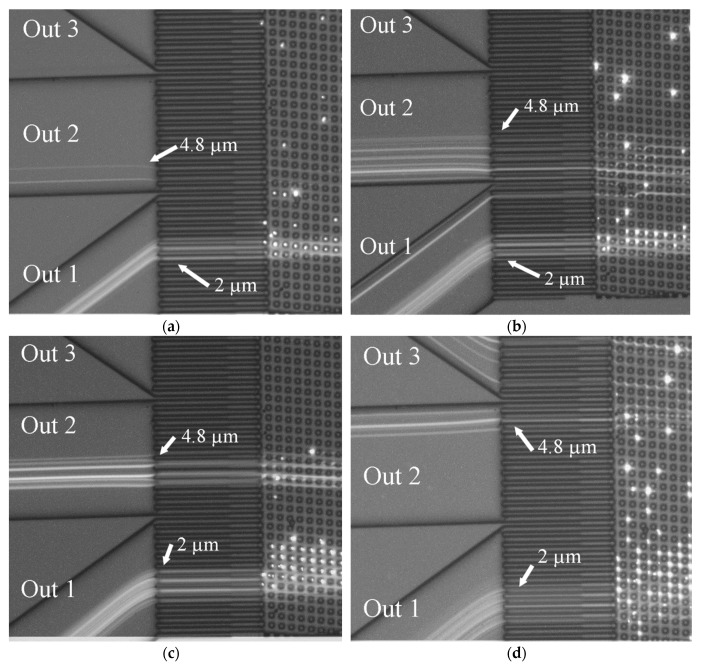
Overlay of 200 video frames with the white light image of the DLD chip areas showing the fractionation position at the end of the array as obtained with fluorescent particles at a size of 2 µm and 4.8 µm for different velocities (Reynolds numbers). (**a**) *Re* = 0.5; (**b**) *Re* = 10; (**c**) *Re* = 15; (**d**) *Re* = 25.

Due to the emerging and growing wake behind the microposts at higher Re, the fractionation behavior of an array will change compared to an operation at lower *Re*. Therefore, particles at a size of 2 µm and 4.8 µm were fed through the sample inlet channel of the array. The fractionation behavior was observed at different *Re* (*Re* = 0.5–25). Due to the design of the array (see also Table 1), we expect the 2 µm particles not to be displaced and therefore remain at the inlet position. Whereas the 4.8 µm particles would be displaced until Section 3 (120 µm wider compared to 2 µm particles) and reach a position at the end of outlet 1 and the beginning of outlet 2. At higher Reynolds numbers, we expect an increase in particle size for each segment caused by the asymmetric flow behind the posts as shown by our PIV measurement and by Dincau et al. [8]. Therefore, the 4.8 µm particles should be displaced further at higher *Re* until they reach outlet 3. Figure 16 shows the sum of 200 consecutive video frames at different Reynolds numbers overlaid with the white light background. The images were taken at 20× magnification. The white dots around the posts in Figure 16 are caused by spontaneous agglomeration around the microposts in individual frames. The probability of bigger particles agglomerating around microposts is lower, therefore posts appear less decorated by particle agglomeration in the fluorescence image. The smaller particles were not visibly affected by the increase of Reynolds numbers and thus remained in Outlet 1. Due to the fluorescence label of the particles, a DLS (Dynamic Light Scattering) measurement of the different outlets was not possible. However, it is visible that the 4.8 µm particles were strongly affected for *Re* > 10, increasing the displacement until they reach outlet 3 at *Re* = 25.

## 4. Conclusions and Outlook

In this work, we successfully designed and fabricated a DLD array consisting of 7 segments to allow fractionation of particles in sub-micrometer steps by changing the tilt angle with a constant gap between the posts. In order to enable operation at higher *Re*, pressure stable systems were realized using processes of dry etching and anodic silicon to glass bonding. CFD simulations predicted growing wakes behind microposts (asymmetric flow) at *Re* > 1. The flow field asymmetry was confirmed for the first time with µPIV measurements using 0.86 µm fluorescent polystyrene particles. The influence of the wakes on particle fractionation was demonstrated using particles at a size of 2 µm and 4.8 µm, which were displaced to slightly different positions at higher *Re*. This confirms that *D_c_* is not only based on array geometry, but can also be influenced by flow velocity The obtained results will be important to understand and optimize high-throughput fractionation using pressure stable DLD devices, which may find interesting applications in post processing of pharmaceutical particulate formulations.

## Figures and Tables

**Table 1:** Variables within each segment to allow 80 µm displacement, tilt angle, length of each segment and resulting critical particle size for fractionation.

Segment	1	2	3	4	5	6	7
Tilt Angle [°]	1	1.6	2.3	3.2	4.2	5.4	6.7
Length [mm]	4.62	2.90	1.98	1.44	1.08	0.86	0.68
*Dc*	3.0	3.8	4.5	5.3	6.0	6.8	7.5

**Table 2:** Input flow rates and velocities in the inlet channels and between the post and Reynolds number.

Q1: Buffer ^1^	Q2: Sample ^1^	Q3: Buffer ^1^	*V_in_* ^2^	*V_post_* ^2^	*Re*
20.01	8.30	146.89	0.194	0.25	3.87
40.02	16.59	293.79	0.387	0.51	7.62
100.04	41.48	734.47	0.968	1.27	19.05
160.07	66.37	1175.16	1.549	2.03	30.48

^1^ All Flow rates in µL/min. ^2^ All velocities in m/s.

**Table 3:** Inlet velocities at different Reynolds numbers.

***V_in_* (cm/s)**	3.8	7.6	19.0	38.1	57.1	76.2	95.2	114.3	133.3	152.4	175.0	200.0	225.0	250.0
***Re***	0.76	1.52	3.81	7.62	11.43	15.24	19.05	22.86	26.67	30.48	35.00	40.00	45.00	50.00

## References

[1] Shekunov B.Y., Chattopadhyay P., Tong H.H.Y., Chow A.H.L. (2007). Particle size analysis in pharmaceutics: Principles, methods and applications. Pharm. Res..

[2] Davis J. (2008). Microfluidic Separation of Blood Components through Deterministic Lateral Displacement. Ph.D. Thesis.

[3] Sajeesh P., Sen A.K. (2014). Particle separation and sorting in microfluidic devices: A review. Microfluid Nanofluid.

[4] Albagdady A., Al-Faqheri W., Kottmeier J., Meinen S., Frey L.J., Krull R., Dietzel A. (2019). Enhanced inertial focusing of microparticles and cells by integrating trapezoidal microchambers in spiral microfluidic channels. RSC Adv..

[5] McGrath J., Jimenez M., Bridle H. (2014). Deterministic lateral displacement for particle separation: A review. Lab Chip.

[6] Wunsch B.H., Smith J.T., Gifford S.M., Wang C., Brink M., Bruce R.L., Austin R.H., Stolovitzky G., Astier Y. (2016). Nanoscale lateral displacement arrays for the separation of exosomes and colloids down to 20 nm. Nat. Nanotechnol..

[7] Lubbersen Y.S., Schutyser M.A.I., Boom R.M. (2012). Suspension separation with deterministic ratchets at moderate Reynolds numbers. Chem. Eng. Sci..

[8] Lubbersen Y.S., Dijkshoorn J.P., Schutyser M.A.I., Boom R.M. (2013). Visualization of inertial flow in deterministic ratchets. Sep. Purif. Technol..

[9] Dincau B.M., Aghilinejad A., Hammersley T., Chen X., Kim J.-H. (2018). Deterministic lateral displacement (DLD) in the high Reynolds number regime: High-throughput and dynamic separation characteristics. Microfluid Nanofluid.

[10] Zeming K.K., Thakor N.V., Zhang Y., Chen C.-H. (2016). Real-time modulated nanoparticle separation with an ultra-large dynamic range. Lab Chip.

[11] Calero V., Garcia-Sanchez P., Honrado C., Ramos A., Morgan H. (2019). AC electrokinetic biased deterministic lateral displacement for tunable particle separation. Lab Chip.

[12] Beech J.P., Tegenfeldt J.O. (2008). Tuneable separation in elastomeric microfluidics devices. Lab Chip.

[13] Loutherback K., D’Silva J., Liu L., Wu A., Austin R.H., Sturm J.C. (2012). Deterministic separation of cancer cells from blood at 10 mL/min. AIP Adv..

[14] Lubbersen Y.S., Fasaei F., Kroon P., Boom R.M., Schutyser M.A.I. (2015). Particle suspension concentration with sparse obstacle arrays in a flow channel. Chem. Eng. Process. Process Intensif..

[15] Dincau B.M., Aghilinejad A., Chen X., Moon S.Y., Kim J.-H. (2018). Vortex-free high-Reynolds deterministic lateral displacement (DLD) via airfoil pillars. Microfluid Nanofluid.

[16] Krüger T., Holmes D., Coveney P.V. (2014). Deformability-based red blood cell separation in deterministic lateral displacement devices—A simulation study. Biomicrofluidics.

[17] Kulrattanarak T., van der Sman R.G.M., Lubbersen Y.S., Schroën C.G.P.H., Pham H.T.M., Sarro P.M., Boom R.M. (2011). Mixed motion in deterministic ratchets due to anisotropic permeability. J. Colloid Interface Sci..

[18] Kulrattanarak T., van der Sman R.G.M., Schroën C.G.P.H., Boom R.M. (2011). Analysis of mixed motion in deterministic ratchets via experiment and particle simulation. Microfluid Nanofluid.

[19] Liu Z., Huang F., Du J., Shu W., Feng H., Xu X., Chen Y. (2013). Rapid isolation of cancer cells using microfluidic deterministic lateral displacement structure. Biomicrofluidics.

[20] Lindken R., Rossi M., Grosse S., Westerweel J. (2009). Micro-Particle Image Velocimetry (microPIV): Recent developments, applications, and guidelines. Lab Chip.

[21] Temiz Y., Lovchik R.D., Kaigala G.V., Delamarche E. (2015). Lab-on-a-chip devices: How to close and plug the lab?. Microelectron. Eng..

[22] Kruusing A. (2004). Underwater and water-assisted laser processing: Part 2—Etching, cutting and rarely used methods. Opt. Lasers Eng..

[23] Olsen M.G., Adrian R.J. (2000). Out of focus effects on particle image visibility and correlation in microscopic particle image velocimetry. Exp. Fluids.

[24] Blevins R.D. (1994). Flow-Induced Vibration.

[25] Kawamura T., Takami H. (1986). Computation of high Reynolds number flow around a circular cylinder with surface roughness. Fluid Dyn. Res..

